# The People Living with HIV (PLHIV) Resilience Scale: Development and Validation in Three Countries in the Context of the PLHIV Stigma Index

**DOI:** 10.1007/s10461-019-02594-6

**Published:** 2019-07-26

**Authors:** A. Gottert, B. Friedland, S. Geibel, L. Nyblade, S. D. Baral, S. Kentutsi, C. Mallouris, L. Sprague, J. Hows, F. Anam, U. Amanyeiwe, J. Pulerwitz

**Affiliations:** 1 Population Council HIV and AIDS Program, Washington, DC USA; 2 Population Council HIV and AIDS Program, New York, NY USA; 3 RTI International, Washington, DC USA; 4 Department of Epidemiology, Johns Hopkins School of Public Health, Baltimore, MD USA; 5 Center for Public Health and Human Rights, Johns Hopkins University, Baltimore, MD USA; 6 National Forum of PLHIV Networks in Uganda (NAFOPHANU), Kampala, Uganda; 7 UNAIDS, Geneva, Switzerland; 8 The Global Network of People Living with HIV (GNP +), Amsterdam, The Netherlands; 9 International Community of WLHIV (ICW), Nairobi, Kenya; 10 Prevention, Care and Treatment (PCT) Division, USAID Office of HIV/AIDS, Washington, DC USA

**Keywords:** Resilience, HIV, PLHIV, Measurement, Stigma

## Abstract

Supporting resilience among people living with HIV (PLHIV) is crucial to their sustained uptake of HIV services as well as psychological and social wellbeing. However, no measures exist to assess resilience specifically in relation to living with HIV. We developed the PLHIV Resilience Scale and evaluated its performance in surveys with 1207 PLHIV in Cameroon, Senegal and Uganda as part of the PLHIV Stigma Index—the most widely used tool to track stigma and discrimination among PLHIV worldwide. Factor analyses demonstrated satisfactory psychometric properties and reliability (alphas = 0.81–0.92). Levels of resilience (e.g., whether one’s self-respect has been positively, negatively, or not affected by one’s HIV status) varied substantially within and across countries. Higher resilience was associated with less depression in each country (all p < 0.001), and, in Cameroon and Uganda, better self-rated health and less experience of stigma/discrimination (all p < 0.001). The final 10-item PLHIV Resilience Scale can help inform interventions and policies.

## Resumen

El apoyo a la resiliencia de las personas que viven con VIH es crucial para la utilización de servicios de salud de VIH como así también su bienestar psicológico y social. Sin embargo, no existen formas para medir la resiliencia específicamente en relación con el VIH. En este estudio desarrollamos la Escala de Resiliencia de la Personas que Viven con VIH y evaluamos su desempeño en encuestas con 1207 personas en Camerún, Senegal y Uganda como parte del Índice de Estigma – la herramienta mas usada para medir estigma y discriminación hacia las personas que viven con VIH en el mundo. El análisis de factores demuestra propiedades psicométricas satisfactorias y confiabilidad (alphas = 0.81-0.92). Los niveles de resiliencia (por ejemplo, si al autorrespeto ha sido positivamente, negativamente o no afectado por el estatus personal de VIH) varió sustancialmente dentro y entre los países. La mayor resiliencia estuvo asociada con menor depresión en cada país (todos p < 0.001), y, en Camerún y Uganda, con mejor autoevaluación de salud y menor experiencias de estigma/discriminación (todos p < 0.001). La Escala final de Resiliencia de Personas que viven con VIH puede contribuir a intervenciones y políticas.

## Introduction

With the expansion of access to highly active anti-retroviral therapy (ART), HIV has evolved from a fatal disease to a manageable chronic condition [1]. The World Health Organization recommends that all people living with HIV (PLHIV) start ART at diagnosis to improve their own health and to reduce the chances of onward transmission [2, 3]. Yet, despite significant scientific advances in prevention and treatment, many people at risk of or living with HIV face barriers to accessing and adhering to treatment that enable long and healthy lives. A growing body of literature has been shifting away from focusing exclusively on predictors of HIV-related morbidity and barriers to health-seeking such as stigma, in favor of an emphasis on understanding and promoting positive factors like “resilience”—both as a means of achieving good health, and as an end in itself [4–6].

The construct of resilience has most often been defined as related to an individual’s “positive adaptation within the context of significant adversity” [4]. Recent theoretical work has built upon this definition by viewing resilience in the face of adversity as depending on the social and structural contexts that surround an individual as much as his or her personal traits or capacities [7]. A 2018 review of the study and definition of resilience in the context of HIV also highlights that in addition to being shaped by contextual forces, resilience itself can be expressed at the individual, collective, or community levels [8]. Similarly, resilience has also been conceptualized as concerning ‘resources’ that draw upon individual capacity, as well as family and community support to overcome adversity [8].

This review also found that the great majority of existing publications did not in fact define resilience at all, so it was not clear what definition was used in a given study. However, based on the measures used for ‘resiliency’ across the available studies, the review authors concluded that there was a need for additional resiliency measures that were tailored for PLHIV; the existing measures examined more general constructs (e.g., social support or a general ability to adapt to change, or to handle pressure) that did not sufficiently capture the issues faced by PLHIV.

Despite the diversity of measures for resilience and the lack of measures specifically tailored for PLHIV, several studies among PLHIV have demonstrated associations between ‘resilience’ (as measured in a given study) and quality of life [9–11], depression [12–17], retention in care and treatment [18–22], and viral load [23]. Many of these studies have used resilience scales that assess general psychological resilience, i.e., not in the context of particular types of adversity. The conner-davidson resilience scale (CD-RISC) [24], a 25-item scale that was originally developed to assess responses to treatment for anxiety, depression and stress reactions, has been most frequently used [11, 12, 15, 16, 18, 23, 25–27]. This scale was primarily oriented around personal qualities that enable thriving in the face of adversity (e.g., “I try my hardest on every occasion” and “I can adapt to change”), but also captures certain features the developers believed to underpin resilience, such as having close personal relationships and experience achieving one’s goals [24]. Other scales used to measure resilience in the existing literature include the brief resilience scale (BRS) [9, 17, 28], the brief resilience coping scale (BRCS) [14, 29, 30], and the dispositional resilience scale [6, 31].

Although the scales described above are generally considered to have good psychometric properties [6], they also have limitations. In particular, the resilience scales described above are not specific to PLHIV. Without measuring resilience explicitly in relation to living with HIV, it is not possible to differentiate it from general abilities or experiences a group of PLHIV respondents may have anyway, independent of their HIV status (e.g., their ability to cope with stress (in general) or have close and secure relationships with others (in general)). This is particularly relevant given the unique nature of living with HIV—a virus that is often acquired through practices already stigmatized in many societies, and for which preventive care is often least available to socially and economically marginalized populations [32].

One exception is the PozQol scale, which is both related to resilience and tailored to assess different aspects of quality of life among PLHIV, such as health concerns, and psychological, social, and functional wellbeing [8]. It was developed in Australia, and derived in part from the BRS. The PozQol scale, while intended for use among PLHIV, combines items specifically related to HIV (e.g., Managing HIV wears me out) with items assessing psychological resilience in general (e.g., I am enjoying life), and also includes items related to perceived effects of HIV on health status (e.g., I worry about the impact of HIV on my health) [9]. Therefore this scale may cover too diverse a range of domains, and with items directly related to HIV and not, to be useful as a measure specifically of resilience related to living with HIV.

To respond to the need for a brief measure to assess resilience specifically in relation to living with HIV, we developed the PLHIV Resilience Scale. We did so in the context of updating the PLHIV Stigma Index. The People Living with HIV Stigma Index (Stigma Index) is a survey implemented by PLHIV among PLHIV. It was developed in 2008 by the Global Network of PLHIV (GNP +), the International Community of Women Living with HIV (ICW), the International Planned Parenthood Federation (IPPF), and UNAIDS to document stigma and discrimination among PLHIV, and to advocate for programs and policies to improve the lives of PLHIV [33, 34]. The Stigma Index was updated in 2017 by a partnership of the index developers (GNP + , UNAIDS, ICW), the Population Council, and other stigma researchers, in response to shifts in the global HIV/AIDS epidemic and changes in treatment guidelines. Based on experiences implementing the Stigma Index in more than 90 countries among more than 100,000 PLHIV, the partners led an iterative process that included consultation with PLHIV networks, advocates and donors [35, 36]. A key recommendation was to add questions about resilience and to incorporate validated measures (scales) wherever possible [34, 36].

In this paper, we describe the development and validation of the PLHIV Resilience Scale. We evaluated the scale using survey data and cognitive interviews in three countries: Cameroon, Senegal and Uganda. HIV prevalence varies across the countries, from 0.4% in Senegal, to 3.7% in Cameroon, to 5.9% in Uganda [37]. We conclude by describing the final scale that resulted from this work, and how it could be applied in the future.

## Methods

### Scale Development Process

Building on the definition of resilience above, we conceptualized resilience among PLHIV as a dynamic process encompassing positive adaptation within the context of living with HIV. ‘Positive adaptation’ implies an improvement in one’s ability to meet a range of physiological, psychological and social needs, for example, as described by Maslow [38]. We therefore structured the Resilience Scale to capture the extent to which one’s ability to meet various needs and important life goals has been affected by having HIV. Further, recognizing that experiencing a significant adversity like having HIV can negatively affect, not affect, or positively affect one’s ability to meet these needs, we included these as the three main response options for each item, as well as “not applicable” and “prefer not to answer” options. The content/topic of items related to self-respect, as well as ability to cope with stress, achieve goals, and have secure relationships with others were drawn from the 25-question CD-RISC [24]. Recognizing that these items tend to reflect personal resources for resilience, and that there may be other more outward-facing life needs/goals that should be captured, we developed other items related to finding love, wanting children, contributing to one’s community, and practicing a religion/faith. We chose a reference period of the last 12 months to minimize recall bias and to facilitate assessment of changes in resilience over time, particularly given that the PLHIV Stigma Index is implemented every few years in many countries. The initial 11-item Resilience Scale was incorporated into the updated PLHIV Stigma Index, and pre-tested among approx. 60 PLHIV in conjunction with the 21st International AIDS Conference (Durban, South Africa 2016), before being formally pilot-tested.

### Study Sample and Data Collection Methods

We validated the Resilience Scale during testing of the updated Stigma Index in Douala and Yaoundé, Cameroon; Dakar and Ziguinchor, Senegal; and greater Kampala, Uganda. These pilot studies were conducted in collaboration with Metabiota and Réseau Cameronais des Associations de Personnes Vivant avec le VIH (RéCAP +) in Cameroon, Enda Santé and Réseau National des Associations de PVVIH du Sénégal (RNP +) in Senegal, and the National Forum of PLHIV Networks in Uganda (NAFOPHANU). In all three countries, we used a combination of two non-probabilistic sampling methods—venue-based and snowball sampling—to enroll a diverse group of PLHIV, and because a complete sampling of PLHIV and PLHIV sub-groups is difficult to establish. Venue-based convenience sampling was employed to recruit PLHIV who are currently linked to care and services via PLHIV networks, community-based organizations (CBOs) serving key populations, or clinics providing HIV care. Snowball sampling, in which study participants invited their peers to be interviewed, was used as a way of including participants who are not currently linked to care and services and might have different experiences of stigma and discrimination than their peers who are linked with care, support, or advocacy groups.

Quantitative survey data were collected through pre-programmed Survey-CTO digital forms on tablet computers (Senegal and Cameroon) or mobile phones (Uganda) in French, Wolof, Diola, Mandingue, Luganda or English. Following standard procedures for the Stigma Index, PLHIV administered the questionnaires to peer respondents living with HIV. The target sample was 400 participants per country.

Informed consent was obtained from all respondents before initiating surveys. The protocol, Stigma Index questionnaire and informed consent forms were approved by the Institutional Review Boards of the Johns Hopkins Bloomberg School of Public Health and the Population Council, and by the Comité National D’Ethique de la Recherche pour la Santé Humaine (Cameroon), the Comité National Ethique pour la Recherche en Santé (Senegal), and the Mildmay Uganda Research Ethics Committee (Uganda).

We evaluated the scale in five steps, described below. First, we used exploratory factor analysis to evaluate the factor structure of this new scale. Second, we used confirmatory factor analysis to test the structural validity of the scale. Third, we examined the scale’s reliability. Fourth, we assessed convergent validity, i.e., whether the scale score was correlated with other theoretically related variables. Fifth, we conducted cognitive interviews with PLHIV to understand their perspectives on the scale and scale items. Based on the results of these steps, we decided on the final set of scale items.

### Factor Analysis

Statistical analyses were carried out separately for the three countries, using Stata v. 15 statistical software [39]. For the Resilience Scale, “not applicable” and “prefer not to answer” responses were recoded as missing. This missing data was < 5%, overall, and < 2% for most items. However, two items in Senegal and Uganda had higher missing data due to ‘not applicable’ responses: “My desire to have children” (16.8 and 6.2% respectively) and “My achievement of my professional goals” (21.2 and 14.7% respectively). In order to conduct complete-case analyses for the exploratory factor analysis (EFA) and confirmatory factor analysis (CFA), we replaced missing values with the mean of individual respondents’ responses on all other items.

We carried out a split-sample EFA/CFA for the set of 11 Resilience Scale items. For each country, we randomly split the sample in half, conducted EFA on the first half to identify the most plausible factor structure and identify any underperforming items, and CFA on the second half to test the structural validity of the structure selected based on the EFA [40]. To determine the factor structure through the EFA, we used the ‘factor’ command in Stata. We examined the extent of the reduction in eigenvalues with each additional factor, then specified a plausible range of number of factors. To determine the factor structure and items to be retained for testing using CFA, we evaluated interpretability (i.e., the extent to which items within any one factor seemed to be tapping into a common theme) and value of factor loadings (with ≥ 0.3 deemed acceptable).

We used CFA to test the factor structure suggested by the EFA and retained items with significant factor loadings (p < 0.05). We then assessed the adequacy of model fit based on commonly recommended cut-off criteria: root mean square error of approximation (RMSEA, with a cu-toff value < 0.06 indicating good fit), the comparative fit index (CFI) and the Tucker–Lewis Index (TLI) (both with cut-offs > 0.95), and the standardized root mean squared residual (SRMR, cut-off < 0.08) [41]. Finally, we reviewed modification indices, added plausible correlated errors [40], re-fit the model, and assessed the adequacy of final model fit using the same cut-off criteria as described above.

### Reliability

We assessed internal consistency reliability of the Resilience Scale by calculating Cronbach’s coefficient alpha as well as Ordinal Theta. Cronbach’s alpha [42] is a standard measure of reliability offered by most statistical software packages. Ordinal theta is a measure of reliability similar to alpha, but based on a polychoric correlation matrix, which is more appropriate than alpha for items with a limited number of ordinal response categories (in this case, three) [43]. For both measures, we interpreted values of ≥ 0.70 to represent adequate reliability and ≥ 0.80 to represent good reliability.

### Convergent Validity

To assess convergent validity, we tested whether the composite Resilience Scale score was associated with three other variables in the updated Stigma Index: overall self-rated health, depression, and experience of stigma/discrimination in the last year. Overall self-rated health was measured by three potential responses (Good, Fair, Poor) to the question, “In general, how would you describe your health at the moment?” Depression was measured by a validated, 4-item version of the Patient Health Questionnaire for Depression and Anxiety (PHQ-4) [44], which in our samples had a Cronbach’s alpha of 0.80 in Cameroon, 0.88 in Senegal and 0.80 in Uganda. Finally, experience of stigma/discrimination in the last year was measured by a binary variable that we created from 11 questions about experiences of stigma and/or discrimination because of HIV status related to social gatherings, family, religious activities, workplace/employment opportunities, verbal harassment, blackmail, physical harassment, and wife/husband/partner having experienced discrimination due to respondent’s HIV status. Respondents who experienced ≥ 1 form of stigma/discrimination in the last year scored a 1 and those who had not scored a 0.

We hypothesized that the Resilience Scale would be positively associated with overall self-rated health, negatively associated with depression, and negatively associated with having experienced stigma/discrimination in the last year. Both bivariate and multivariate regression analyses were employed; multivariate analyses controlled for age, gender identity, and education.

### Cognitive Interviews

In addition to the quantitative data collection, cognitive interviews were conducted among 20 respondents per country (60 total) to explore respondents’ perceptions of the importance of question topics and understanding of the meaning of and reactions to specific questions [45], with a focus on questions that had been added to or modified from the original PLHIV Stigma Index. In Uganda and Cameroon, cognitive interviews were conducted with 20 participants who had not participated in the quantitative survey, whereas in Senegal, a subset of 20 respondents from the quantitative survey were asked to participate in cognitive interviews after quantitative data collection was completed. In each country, we sought a diverse sample of cognitive interview participants in terms of demographics and key population status, and recruited participants using similar methods as for the main surveys. During the cognitive interviews, respondents were asked their opinions about the importance of the resilience questions, whether they agreed with the specific items included, whether they thought there were any items that were missing, and if they felt the timeframe of the questions (in the last 12 months) was relevant for the specific items. Informed consent to participate in the cognitive interviews was obtained from all participants.

## Results

A total of 1207 respondents completed the quantitative survey. When asked how they would describe themselves, a majority said female, and between two and six percent reported being transgender (Table 1). Respondents in each country also reported currently or ever belonging to the following groups: MSM (just under 10%), female sex workers (15–30%), and persons who inject drugs (2–5%). Mean age was late 30 s to early 40 s, and respondents had known their HIV-positive status, on average, for at least 7 years. A minority of respondents had completed a secondary education. In terms of disclosure status, nearly all respondents in each country believed that at least one referent group (e.g., husband/wife/partner, friends, co-workers, etc.) knew their HIV status). Most respondents in each country said their overall health was good or fair. The mean depression score was lowest in Uganda and highest in Cameroon. Over half (55%) of respondents in Cameroon reported experiencing at least one type of stigma/discrimination in the last year, versus 36% in Uganda and 15% in Senegal.

**Table 1:** Sample characteristics, by country

	Cameroon (n = 400)	Senegal (n = 406)	Uganda (n = 401)
Gender identity			
Female	72.2%	79.0%	59.9%
Male	25.3%	20.1%	34.2%
Transgender	2.5%	1.0%	6.0%
Age—mean (range)	37.9 (18–69)	42.1 (18–70)	36.2 (18–81)
Years knowing status—mean (range)	7.8 (0–27)	12.6 (0–23)	6.8 (0–58)
Completed secondary/high school (vs. less)	39.3%	18.6%	17.0%
Key population status			
Men who have sex with men (MSM)^a^	9.8%	9.6%	7.5%
Female sex worker	15.3%	18.7%	28.7%
Person who injects drugs	1.5%	2.2%	5.2%
HIV status disclosed to others^b^	99.5%	98.0%	89.8%
Overall self-rated health			
Poor	11.0%	2.7%	5.0%
Fair	43.8%	48.2%	27.9%
Good	45.3%	49.1%	67.1%
Depression—mean (SD) (range of 1.0 to 4.0)	2.3 (0.87)	1.8 (0.82)	1.5 (0.65)
Experienced stigma/discrimination in last year	55.3%	14.5%	36.3%

^a^About half of respondents in each country who reported being transgender also reported MSM key population status

^b^Respondent believes at least one referent group (e.g., husband/wife/partner, friends, co-workers, etc.) knows his or her HIV status

### Descriptive Statistics—Resilience Scale Items and Composite Score

In each country, when asked to rate the extent to which having HIV has affected various aspects of their quality of life, a majority of respondents answered “not affected,” with sizeable minorities answering, *“*positively affected” or “negatively affected” (Table 2). There was substantial variation (SD 4.4–4.8) in scores within each country, and scores spanned the possible range (− 11 to + 11). In general, Cameroon had the largest proportion of respondents answering “negatively affected” across items—over 50% for many items.

**Table 2:** PLHIV Resilience Scale item frequencies and scale scores, by country

Item	Cameroon (n = 400) %			Senegal (n = 406) %			Uganda (n = 401) %		
	+	Not aff.	–	+	Not aff.	–	+	Not aff.	–
Please answer whether your ability to meet your needs in the last 12 months has been positively affected, not affected, or negatively affected, by your HIV status									
a. My self-confidence	14.8	31.6	53.6	19.7	61.8	18.5	27.0	54.0	19.0
b. My self-respect	15.8	45.0	39.3	18.5	71.1	10.4	25.3	65.8	9.0
c. My ability to respect others	17.1	64.3	18.6	15.3	80.1	4.7	24.9	72.9	2.3
d. My ability to cope with stress	16.5	35.6	47.9	18.8	64.1	17.1	23.9	55.2	20.9
e. My ability to have close and secure relationships with others	9.8	42.0	48.2	16.9	72.5	16.9	22.4	62.9	14.8
f. My ability to find love	8.5	32.1	59.5	12.9	65.4	21.6	18.6	56.9	24.5
g. My desire to have children	9.0	35.6	55.4	16.6	57.5	25.9	14.1	54.0	32.0
h. My achievement of my personal goals	14.9	38.3	46.9	15.1	69.1	15.8	20.9	50.9	28.2
i. My achievement of my professional goals	15.6	40.0	44.4	10.4	73.4	16.2	25.9	52.1	22.0
j. My ability to contribute to my community	19.9	54.7	25.5	14.9	76.0	9.1	24.4	64.4	11.3
k. My ability to practice a religion/faith as I want to	25.9	58.4	15.7	17.4	79.9	2.7	28.4	65.8	5.8
Mean composite scale score (SD, range)	− 2.9 (4.4, − 11 to + 11)			0.34 (4.4, − 11 to + 11)			0.69 (4.8, − 11 to + 11)		

Correspondingly, the mean of the composite scale scores was substantially lower in Cameroon than in Senegal or Uganda, where the mean scores were slightly more positive than negative. We conducted ancillary analyses to clarify whether there were associations between mean resilience scores and two variables related to HIV status (controlling for other demographic characteristics): years knowing status, and others knowing one’s HIV status. For years knowing status, there was no association in Cameroon or Senegal, but a positive association in Uganda, that is, the longer respondents had known their status, the higher their resilience scores (Beta = 0.18 (95% CI 0.10, 0.26), p < 0.001) (data not shown). In terms of other people in respondents’ lives knowing their status, since this was so common (over 90% in each country), we could not ascertain whether people whose status is undisclosed have higher or lower resilience than those whose status is disclosed.

### EFA, CFA & Reliability Results

In EFAs, eigenvalues fell dramatically between the first and second factors in all three countries, from > 3 for factor 1 to < 1 for factor 2. This suggested that a one-factor solution was most suitable. Factor loadings for the 11 items were all > 0.4 in Cameroon, > 0.5 in Senegal, and > 0.3, in Uganda. This suggested that all items should be retained for testing in CFAs. In CFAs testing a unidimensional model with the 11 items, all factor loadings (Table 3) were statistically significant at p < 0.001 and therefore all items were retained. Nearly all loadings were above 0.5, with the exception of three items in Cameroon with loadings between 0.3 and 0.5. Fit statistics for models that incorporated correlated error terms met most pre-established cut-off criteria of RMSEA < 0.06, CFI and TLI > 0.95, and SRMR < 0.08 (specific fit statistics by country are available from the authors). The RMSEA came close to meeting fit criteria in all three countries (0.06–0.08), and the TLI came close in Cameroon and Uganda (0.93 and 0.89, respectively). All other cut-off criteria were met. Taken together, these fit statistics suggest adequate model fit in each country.

**Table 3:** Item factor loadings from CFA, by country

Item	Factor loading (Cameroon)	Factor loading (Senegal)	Factor loading (Uganda)
a. My self-confidence	0.44	0.73	0.52
b. My self-respect	0.45	0.80	0.53
c. My ability to respect others	0.41	0.64	0.45
d. My ability to cope with stress	0.42	0.73	0.50
e. My ability to have close and secure relationships with others	0.40	0.76	0.44
f. My ability to find love	0.50	0.65	0.46
g. My desire to have children	0.34	0.68	0.41
h. My achievement of my personal goals	0.73	0.75	0.75
i. My achievement of my professional goals	0.77	0.79	0.73
j. My ability to contribute to my community	0.60	0.73	0.55
k. My ability to practice a religion/faith as I want to	0.30	0.59	0.30

All factor loadings were significant at p = 0.001

CFA sample sizes were 200 in Cameroon, 203 in Senegal, and 200 in Uganda

Internal consistency reliability of the scale was very good in each country. Cronbach’s coefficient alpha was 0.81 in Cameroon, 0.92 in Senegal, and 0.89 in Uganda. Ordinal theta was 0.87 in Cameroon, 0.95 in Senegal, and 0.95 in Uganda.

### Convergent Validity—Associations with Other Variables of Interest

As shown in Table 4, higher Resilience Scale score was significantly associated with higher overall self-rated health in Cameroon and Uganda (both p < 0.001), but was not associated in Senegal. Higher depression score was significantly associated with lower Resilience Scale score in all three countries (all p < 0.001). Finally, having experienced stigma/depression in the last year was significantly associated with lower Resilience Scale score in Cameroon and Uganda (both p < 0.001), but was not associated in Senegal.

**Table 4:** Bivariate and multivariate regression results for PLHIV resilience scale score, by country

	Cameroon (n = 400)		Senegal (n = 406)		Uganda (n = 401)	
Variable	Bivariate	Mulitvar.	Bivariate	Mulitvar.	Bivariate	Mulitvar.
Overall self-rated health	1.66***	1.68***	− 0.28	− 0.34	1.56***	1.63***
Depression score	− 1.15***	− 1.05***	− 1.05***	− 1.00***	− 1.68***	− 1.73***
Experienced stigma/discrimination in last year	− 1.67***	− 1.58***	− 0.65	− 0.57	− 2.18***	− 2.14***

All values presented are beta coefficients. Multivariate analyses controlled for age, gender identity, and education

*p < 0.05, **p < 0.01, ***p < 0.001

### Cognitive Interviews

In cognitive interviews, nearly all respondents said the Resilience Scale questions were important, as illustrated by the following quotes:

“These questions are important because they show that we can play an important role in society and that being HIV-positive is not a fatality.” 59-year-old woman, Cameroon

“These questions are important because it shows that as PLHIV/MSM (men who have sex with men), we can contribute positively to our society.” 23-year-old man, Senegal

When asked about their reactions to the set of items, nearly all felt that each of the items was appropriate and that the list was complete. As one 50-year old Senegalese man pointed out, “PLHIV have the same desires as other people.” Five respondents suggested additional items (cooking, n = 1; sports/exercise, n = 2; relationship with spouse, n = 1; ability to travel, n = 1). In Uganda, many respondents did not see enough of a distinction between the item asking about ability to meet personal goals and the item asking about ability to meet professional goals. For example, a 20-year old male said a personal goal is “something you feel like reaching to, having it or benefitting from—like building a big orphanage centre, being a sports anchor, being a football coach and a good father.”

### Final Scale

**Fig. 1:**
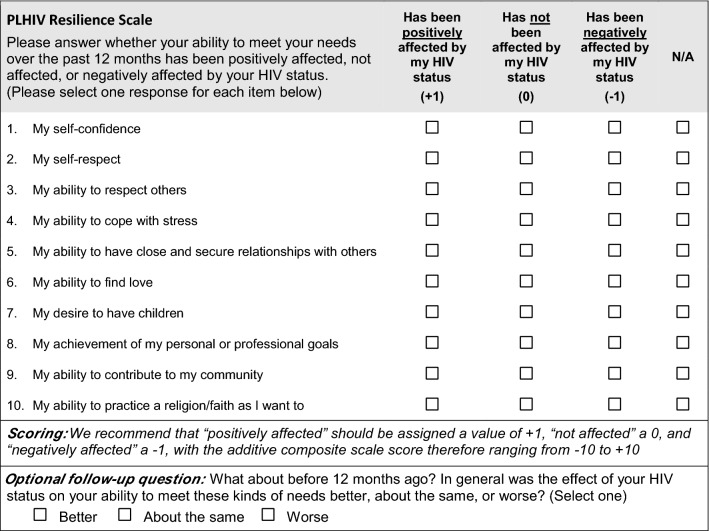
Final PLHIV resilience scale

The analyses described above resulted in a final 10-item scale (Fig. 1), in which we kept nine items the same but combined personal and professional goals into one item. Combining these two items was supported both by cognitive interview data as well as the relatively high percentage of respondents (~ 15–20% in Senegal and Uganda) who selected “not applicable” to the question about professional goals. The possible range of the final composite scale score is − 10 to + 10. To calculate the composite score, we recommend taking the mean of all non-missing items, then multiplying by 10. Since the scale only asks about the last 12 months, it may be beneficial to include an optional survey question following the scale to measure self-assessed change in resilience compared with over a year ago (Fig. 1); this question was not included in the pilot surveys but is currently included in the updated Stigma Index.

## Discussion

Developing a measure of resilience specifically in the context of living with HIV is critical to designing effective interventions and policy efforts to promote health and well-being among PLHIV. Supporting the uptake of and adherence to antiretroviral treatment is particularly important in light of the impact viral load suppression can have on transmission at a population level [46]. The new PLHIV Resilience Scale performed well in factor analyses, demonstrated very good reliability, and captured a range of experiences of resilience both within and between countries. Further, significant associations between the scale and overall health, depression, and experience of stigma/discrimination support convergent validity. Results from the EFA and CFA served to confirm the performance of all 11 items, and the cognitive interviews suggested combining two items into one, resulting in a final 10-item scale. The new PLHIV Resilience Scale (Fig. 1) is now included in the PLHIV Stigma Index 2.0.

Within each country, individuals’ composite scale scores (sum of all items) varied widely, suggesting that the scale can capture a diverse range of resilience experiences. In addition, each scale item exhibited a good range of responses: while a majority of respondents tended to answer ‘was not affected by my HIV status’, a sizeable minority answered either ‘was positively affected’ or ‘was negatively affected’. It was surprising to us that most respondents answered ‘was not affected’ for most items. It is possible that the recall period of the last 12 months (vs. ever, for example) could have decreased the likelihood that having HIV had affected the particular item topic in a meaningful way. This finding could also reflect improvements in HIV treatments and consequent decreases in HIV-related morbidity, which may have reduced the extent to which having HIV is salient to different aspects of people’s lives. Of note, while in Senegal and Cameroon levels of resilience did not differ by years knowing status, in Uganda a longer time knowing status as associated with greater resilience. Since nearly all respondents reported that others knew their HIV status, we were unable to determine whether those who do not disclose are more (or less) likely to be resilient. Associations between resilience and particular referent groups knowing one’s status (e.g., partner vs. other family vs. community), and respondents’ experiences disclosing to these groups, will be important to explore in future research. Finally, it was also notable to us that such a sizable minority of respondents answered that they were positively affected by their HIV status; this serves to further reaffirm the importance of capturing positive, and not only negative, effects of HIV status on individuals’ wellbeing [47, 48].

Across the three countries, responses also varied substantially for each item. In addition, the mean of individuals’ scores varied between the three countries, with Cameroon exhibiting the lowest levels of resilience (mean score was less than zero, i.e., more negatively affected than positively), and Uganda the highest (mean score was less than zero, i.e., more positively affected than negatively). This suggests that the Resilience Scale can capture differences in resilience at a population level, which is important for informing policy and advocacy efforts. It appears that PLHIV are experiencing the lowest levels of resilience in the Cameroonian context and the highest levels of resilience in the Ugandan context. We intend to explore possible reasons for these differing levels of resilience in subsequent analyses of the Stigma Index 2.0 data, which, alongside experiences of HIV-related stigma and discrimination, include variables related to living situation, disclosure experiences, interactions with health services, human rights and effecting change, and stigma and discrimination for reasons other than HIV status (e.g., key population membership). To the extent possible, future research that incorporates the PLHIV Resilience Scale (which taps into resilience at the individual level) should seek to assess social and structural factors external to the individual that may shape individuals’ resilience experiences over time.

Another important area for future exploration is how well the new scale can track changes in resilience over time, within individuals or at the population level. The Stigma Index (both the previous version, and as planned for the Stigma Index 2.0) is often implemented every few years in a given country to track trends over time—providing an important opportunity to see whether the Resilience Scale can document changes in resilience over time, including in response to interventions, advocacy efforts, and policy changes.

Finally, in bivariate and multivariate analyses assessing associations with health status, depression and experience of stigma, a majority of associations were highly significant and in the hypothesized directions. Higher resilience was associated with less depression in all three countries, and, in Cameroon and Uganda, better self-rated health and less experience of stigma/discrimination. In Senegal, however, resilience was not associated with health status or experience of stigma. The proportion of respondents who had experienced recent stigma/discrimination was lower in Senegal (14%) than Uganda or Cameroon, which may have limited our ability to find significant associations with resilience in that country. Taken together, these findings support convergent validity, i.e., that the scale is associated with other theoretically related variables. As described previously, more general resilience scales like the CD-RISC have demonstrated associations with mental and physical health outcomes in other studies among PLHIV [12, 15, 16]. However, it remains unclear the extent to which resilience as measured in those studies was in relation to HIV status—and hence what, precisely, was associated with those outcomes. The significant associations in our study also suggest that improving resilience among PLHIV could improve public health outcomes of interest, such as depression and overall health.

Another area for future research is how resilience fits into the picture of PLHIV stigma and discrimination, and how it relates to health outcomes like HIV treatment uptake or viral load suppression. For example, does resilience directly influence HIV-related health outcomes, as some previous studies have suggested [12, 18, 49], or does it serve to buffer the adverse effect of stigma on health outcomes (or both)? Moreover, can interventions improve PLHIV resilience (and if so, does this lead to improved health outcomes)? Alongside interventions to address stigma and discrimination [50–52], existing interventions related to promoting resilience among PLHIV focus on building coping skills to better manage the effects of this stigma [53], enhancing stress management [54], and increasing social support [29, 47]. The Resilience Scale could aid in further evaluating and strengthening these types of interventions, as well as developing other efforts, including structural changes to promote resistance and empowerment in the face of stigma [5].

This study has several limitations. First, the Resilience Scale focuses on experiences at the individual level (as do most resilience scales). With recent theoretical discussions concerning how to broaden this perspective to include social and structural influencers and/or expressions of resilience, it may be useful to include related items in potential future administrations of the scale. Second, the Scale was tested in comparatively limited samples in three sub-Saharan African countries, and findings may be different in other contexts. Further, although the sample of PLHIV in each country included important sub-groups of ‘key populations’ such as MSM, transgender individuals, and sex workers, it was beyond the scope of this paper to present findings by sub-group Third, it is unclear whether the scale, if administered independent of the full Stigma Index instrument, would perform differently than when administered as part of Index. Finally, because data used in regression analyses were cross-sectional, it is not possible to be sure of temporality of associations; for example, it could be that improved health or depression improves individuals’ resilience rather than the other way around. Each of the limitations just described is fertile ground for future research.

## Conclusion

The People Living with HIV Stigma Index 2.0 [34], which now includes the 10-item Resilience Scale, is anticipated to be implemented in multiple countries in the coming years, yielding additional evidence of the scale’s performance and valuable insight into quality of life of PLHIV. Investigators can also choose to use the scale in other studies with PLHIV, independent of the Stigma Index. Taken together, our results suggest that the Resilience Scale can measure resilience among PLHIV, facilitating the tracking of change over time, as well as informing interventions, policies and advocacy efforts.
